# Complete Genome Sequences of Three Invasive Strains of Streptococcus pyogenes Subtype *emm*5.23 Isolated in Scotland

**DOI:** 10.1128/MRA.00101-21

**Published:** 2021-04-15

**Authors:** Davide Pagnossin, Andrew Smith, William Weir, Chiara Crestani, Diane Lindsay, Roisin Ure, Katarina Oravcova

**Affiliations:** a Institute of Biodiversity, Animal Health and Comparative Medicine, University of Glasgow, Glasgow, United Kingdom; b School of Veterinary Medicine, University of Glasgow, Glasgow, United Kingdom; c Bacterial Respiratory Infection Service, Scottish Microbiology Reference Laboratory, Glasgow Royal Infirmary, Glasgow, United Kingdom; d College of Medical, Veterinary & Life Sciences, Glasgow Dental Hospital & School, University of Glasgow, Glasgow, United Kingdom; University of Maryland School of Medicine

## Abstract

Streptococcus pyogenes *emm*5.23 is uncommon; however, it has recently been involved in a relatively high proportion of cases of invasive disease in Scotland. Here, we report the complete genome sequences of three *emm*5.23 isolates, which may be used as a reference for investigating the virulence and epidemiology of this strain.

## Abstract (precis)

Streptococcus pyogenes *emm*5.23 is uncommon; however, it has recently been involved in a relatively high proportion of cases of invasive disease in Scotland. Here, we report the complete genome sequences of three *emm*5.23 isolates, which may be used as a reference to investigate the virulence and epidemiology of this strain.

## ANNOUNCEMENT

Streptococcus pyogenes is a Gram-positive human pathogen that can cause superficial or invasive infections (1). S. pyogenes strains are classified as *emm* types and subtypes based on the sequence identity of the first 180 bp of the *emm* gene (https://www.cdc.gov/streplab/groupa-strep/emm-background.html). Here, we report the closed genomes of three invasive isolates of S. pyogenes *emm*5.23 collected in Scotland in 2015, 2018, and 2019. This *emm* subtype has been circulating in Scotland since 2015, causing a relatively high proportion of invasive disease cases. In 2018 and 2019, *emm*5.23 was, respectively, the sixth and the second most common strain involved in S. pyogenes invasive disease in Scotland (4.7% and 9.8% of all cases). To our knowledge, *emm*5.23 strains are not commonly isolated worldwide (2) but have been reported in one outbreak of invasive disease (3) and in association with increased mortality in the United Kingdom (4). The three closed and annotated genomes we present here may be utilized as references for mapping-based sequencing studies, which may in the future elucidate why this *emm* subtype is disproportionately responsible for invasive infection in Scotland.

Invasive S. pyogenes strains isolated in Scotland are routinely collected and *emm* typed by the Bacterial Respiratory Infection Service of the Scottish Microbiology Reference Laboratory (SMiRL) in Glasgow, United Kingdom. In order to produce complete and closed whole-genome sequences (WGS) for our three isolates, we used a hybrid assembly approach, merging short Illumina reads and long Oxford Nanopore reads. Illumina sequencing was performed at the SMiRL, while Oxford Nanopore Technologies (ONT) (Oxford, UK) MinION sequencing was carried out at the University of Glasgow. Prior to DNA extraction, a single colony of each strain was inoculated in Todd-Hewitt broth (THB) (Thermo Fisher Scientific, Waltham, MA) for overnight growth at 37°C. For Illumina sequencing, bacterial cells were lysed using mutanolysin, lysozyme, and proteinase K. Genomic DNA was extracted with the DNeasy 96 blood and tissue kit (Qiagen, Hilden, Germany) according to the manufacturer’s instructions. Extracted DNA was quantified using a Qubit 3 fluorometer (Thermo Fisher, UK), and sequencing libraries were generated using the NexteraXT DNA sample preparation kit and index kit v2 (Illumina, San Diego, CA). Next-generation paired-end WGS was then performed using Illumina MiSeq technology. Paired-end raw reads in FASTQ format were trimmed using ConDeTri v3.11.1 (5) with default settings. The script FilterPCRdupl (5) was then used to remove redundant read copies that might have emerged in the PCR step. For Oxford Nanopore MinION sequencing, bacterial cells were lysed using lysozyme and proteinase K, and genomic DNA was extracted using the Wizard genomic DNA purification kit (Promega, Madison, WI, USA). Genomic DNA was quantified with both a Qubit 3 fluorometer and a Nanodrop spectrophotometer, and the MinION input concentration was adjusted to approximately 57 ng/μl. Libraries were prepared using the rapid barcoding kit SQK-RBK004 vRBK_9054_v2_revQ_14Aug2019 (Oxford Nanopore Technologies) following the manufacturer’s instructions. The genomic libraries were sequenced using a MinION flow cell v9.4.1 on a MinION sequencer (Oxford Nanopore Technologies). The raw read files were base called and demultiplexed with Guppy v3.6.0 (6). The genomes were *de novo* assembled using Unicycler v0.4.8 (7) in the hybrid assembly mode, merging the Illumina and MinION reads. Unicycler was used to confirm that complete circular genomes had been generated and to rotate each genome so that it commenced with the *dnaA* gene encoded on the forward strand. Annotations were produced using the NCBI Prokaryotic Genome Annotation Pipeline v5.0 (8).

The sequence characteristics of the three *emm*5.23 genomes are presented in Table 1.

**TABLE 1:** Isolation, sequencing data, and genome characteristics of the three *S. pyogenes* *emm*5.23 isolates

Characteristic^*a*^	Data for isolate^*b*^:		
	iGAS376	iGAS391	iGAS426
Yr of isolation	2018	2019	2015
Origin	Edinburgh, Scotland	Glasgow, Scotland	Glasgow, Scotland
Source	Blood	Blood	Blood
No. of Illumina reads (pairs)	731,618	572,775	647,143
No. of sequenced bp, Illumina	190,745,659 forward, 188,292,105 reverse	145,710,885 forward, 148,445,698 reverse	161,008,620 forward, 163,918,397 reverse
Genome coverage, Illumina (×)	100	78	86
No. of ONT reads	28,201	95,719	90,232
ONT read length *N*_50_ (bp)	14,576	9,633	8,828
No. of sequenced bp, ONT	192,673,926	451,467,050	379,063,977
Genome coverage, ONT (×)	102	238	198
Genome size (bp)	1,897,124	1,897,129	1,897,111
GC content (%)	38.6	38.6	38.6
No. of genes	1,913	1,913	1,913
No. of CDSs	1,824	1,824	1,824
Coding genes	1,775	1,775	1,775
rRNAs (5S, 16S, 23S)	6, 6, 6	6, 6, 6	6, 6, 6
tRNAs	67	67	67
MLST	ST99	ST99	ST99
GenBank accession no.	CP067010	CP067009	CP067008

a CDS, coding DNA sequences; MLST, multilocus sequence type.

b ST99, sequence type 99.

### Data availability.

The raw reads and whole-genome assemblies are available under the NCBI BioProject accession number PRJNA685009, and annotation files are available under the GenBank accession numbers CP067008, CP067009, and CP067010.
